# Supplementary material for: High-performance pipeline for MutMap and QTL-seq
Source: PeerJ. 2022 Mar 18;10:e13170. doi: 10.7717/peerj.13170 (PMC8935991; doi:10.7717/peerj.13170)
Supplement: Figure S1 — (A) MutMap plot of Hit1917-pl from MutMap v1.4.5. (B) MutMap plot of Hit1917-pl from MutMap v2.3.2. (C) MutMap plot of Hit1917-sd from MutMap v1.4.5. (D) MutMap plot of Hit1917-sd from MutMap v2.3.2. (E) QTL-seq plot of RILs from QTL-seq v1.4.5. (F) QTL-seq plot of RILs from QTL-seq v2.2.2. (G) QTL-seq plot of F2 progeny from QTL-seq v1.4.5. (H) QTL-seq plot of F2 progeny from QTL-seq v2.2.2. [file peerj-10-13170-s001.pdf]

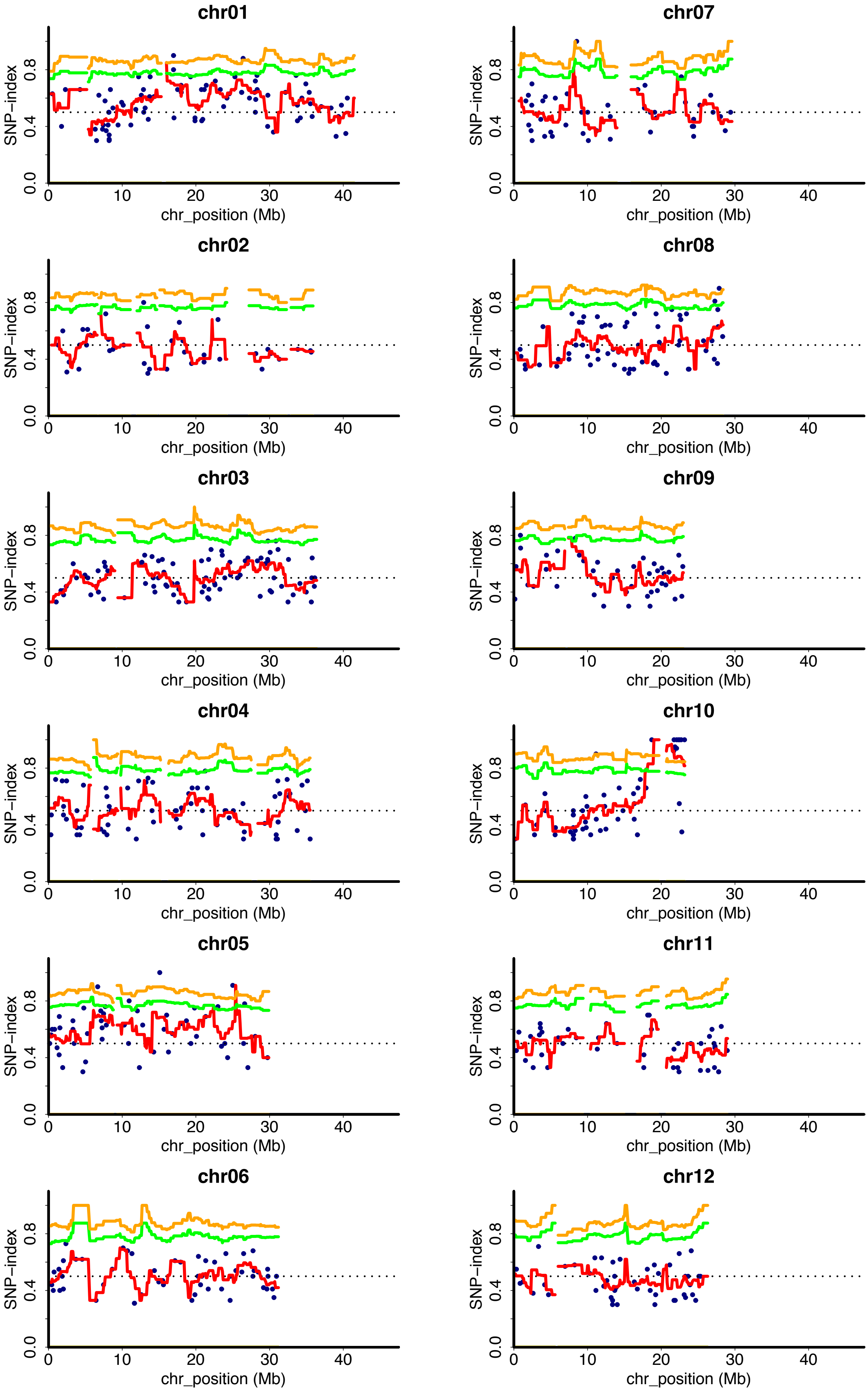

Supplemental Figure S1 (A) MutMap plot of Hit1917-pl from MutMap v1.4.5. Statistical confidence intervals under the null hypothesis of no QTL are shown (green: $P < 0.05$; yellow: $P < 0.01$).

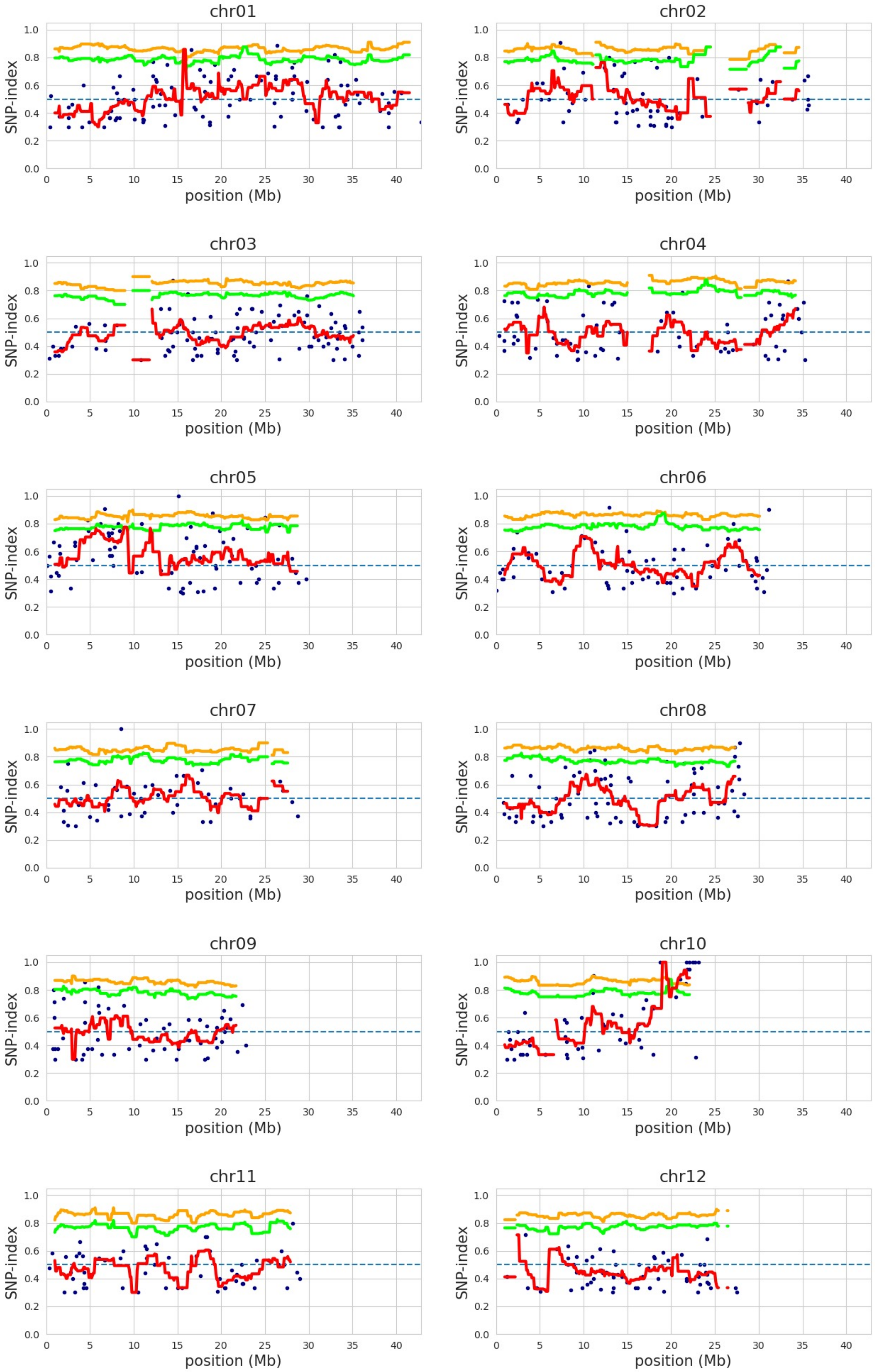

Supplemental Figure S1 (B) MutMap plot of Hit1917-pl from MutMap v2.3.2. Statistical confidence intervals under the null hypothesis of no QTL are shown (green: $P < 0.05$; yellow: $P < 0.01$).

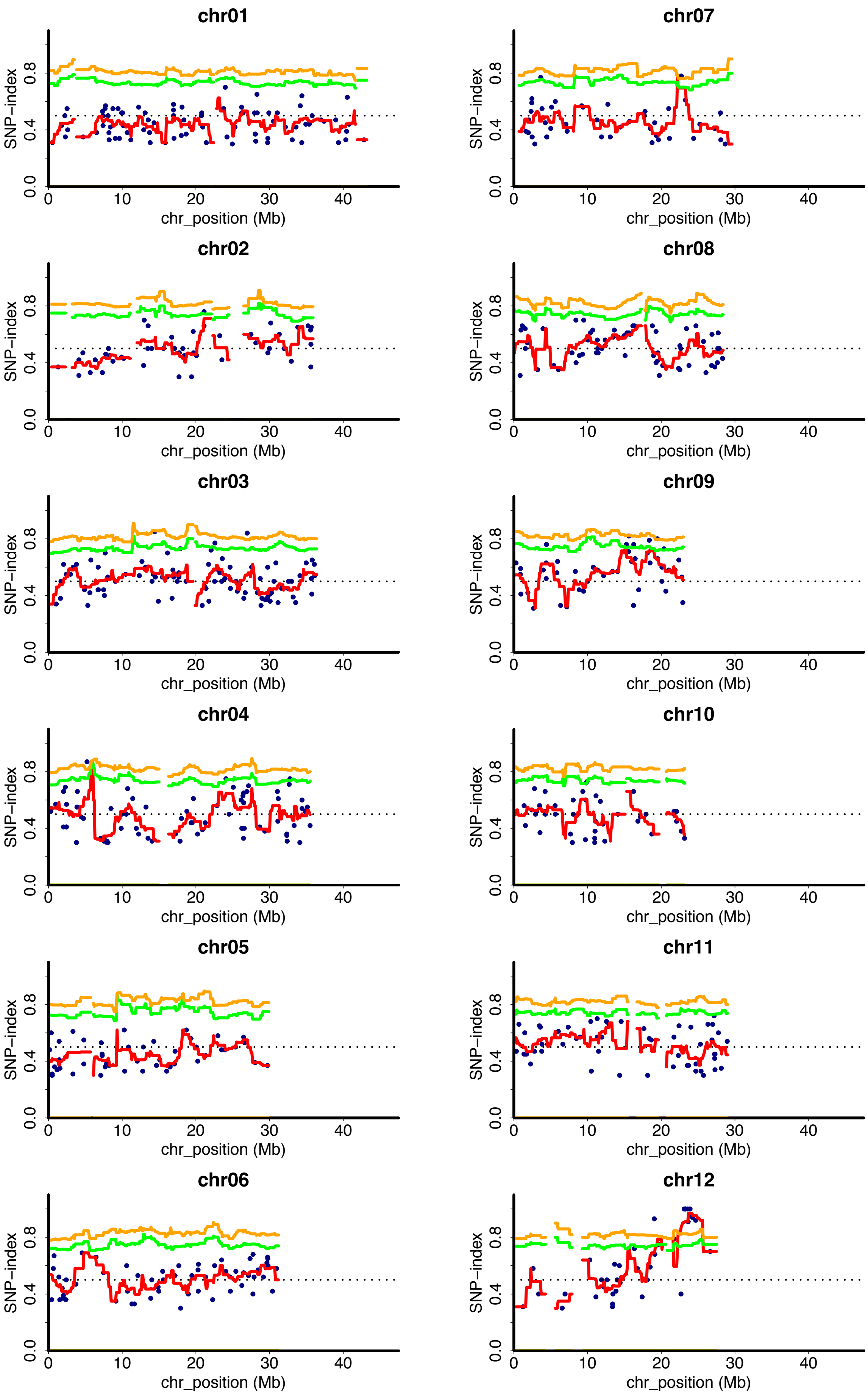

Supplemental Figure S1 (C) MutMap plot of Hit1917-sd from MutMap v1.4.5. Statistical confidence intervals under the null hypothesis of no QTL are shown (green: $P < 0.05$; yellow: $P < 0.01$).

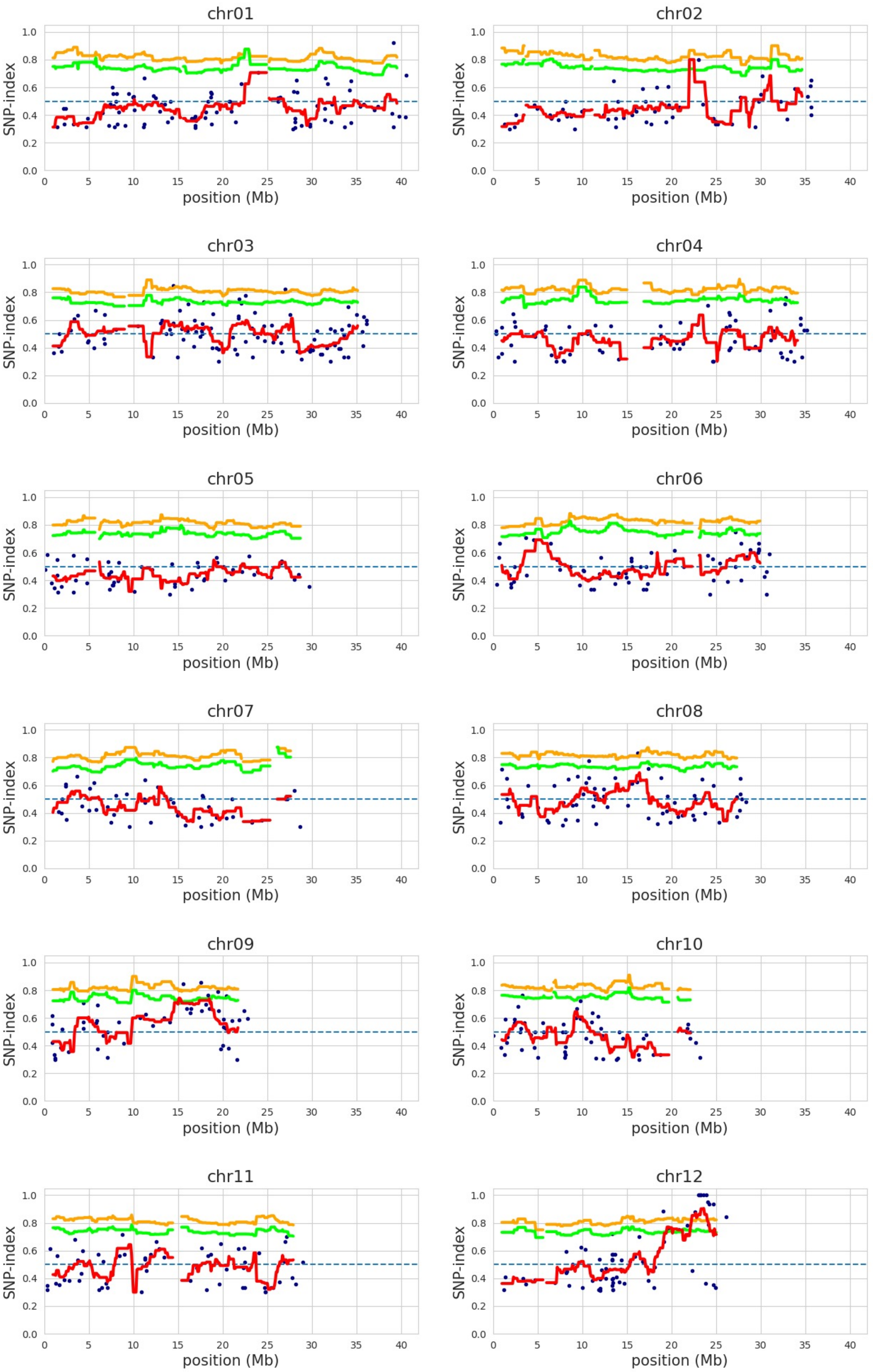

Supplemental Figure S1 (D) MutMap plot of Hit1917-sd from MutMap v2.3.2. Statistical confidence intervals under the null hypothesis of no QTL are shown (green: $P < 0.05$; yellow: $P < 0.01$).

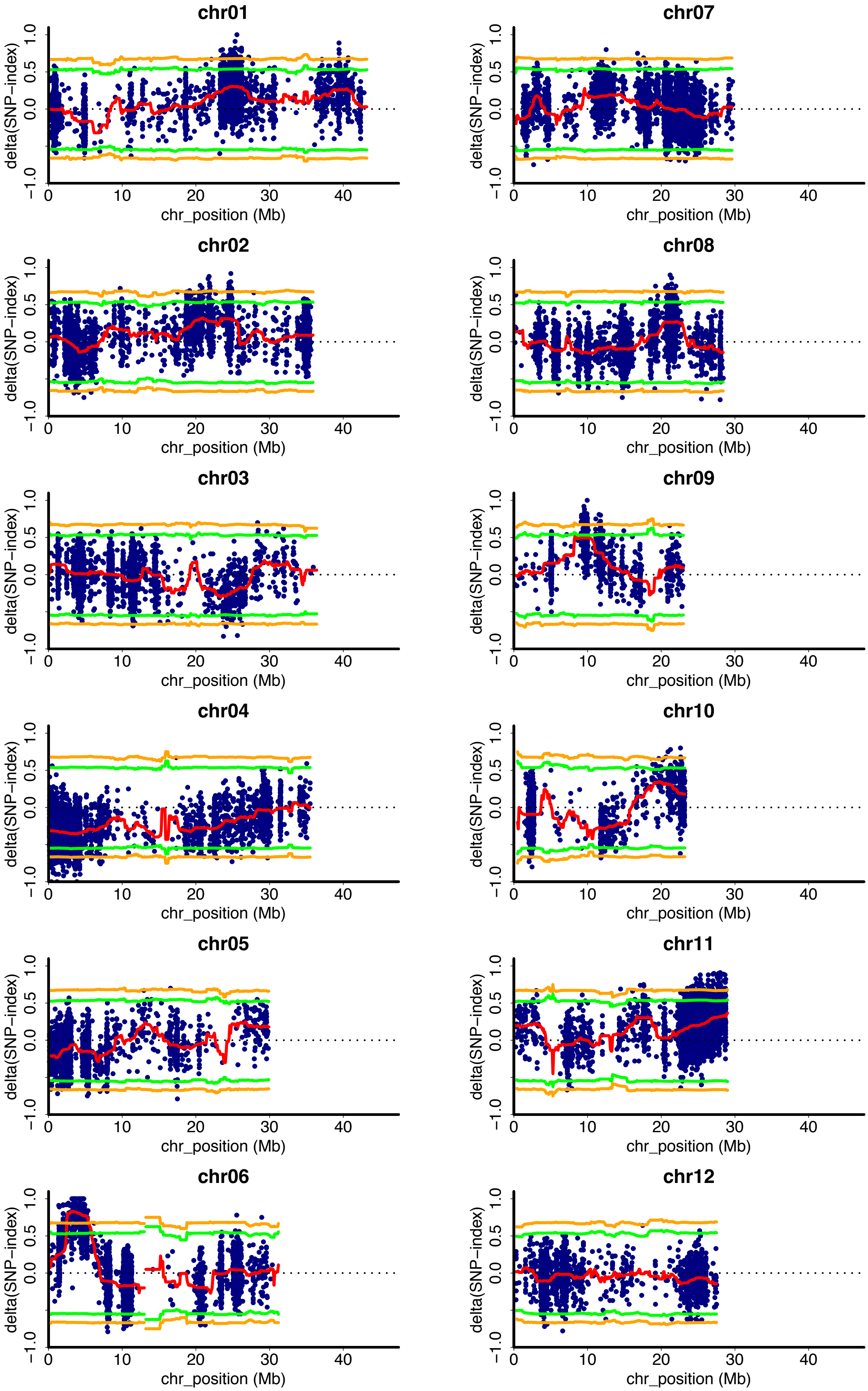

Supplemental Figure S1 (E) QTL-seq plot of RILs from QTL-seq v1.4.5.
The ΔSNP-index plot obtained by subtraction of the susceptible-bulk SNP-index from the resistance-bulk SNP-index for RILs obtained from a cross between Nortai and Hitomebore. Statistical confidence intervals under the null hypothesis of no QTL are shown (green: $P < 0.05$; yellow: $P < 0.01$).

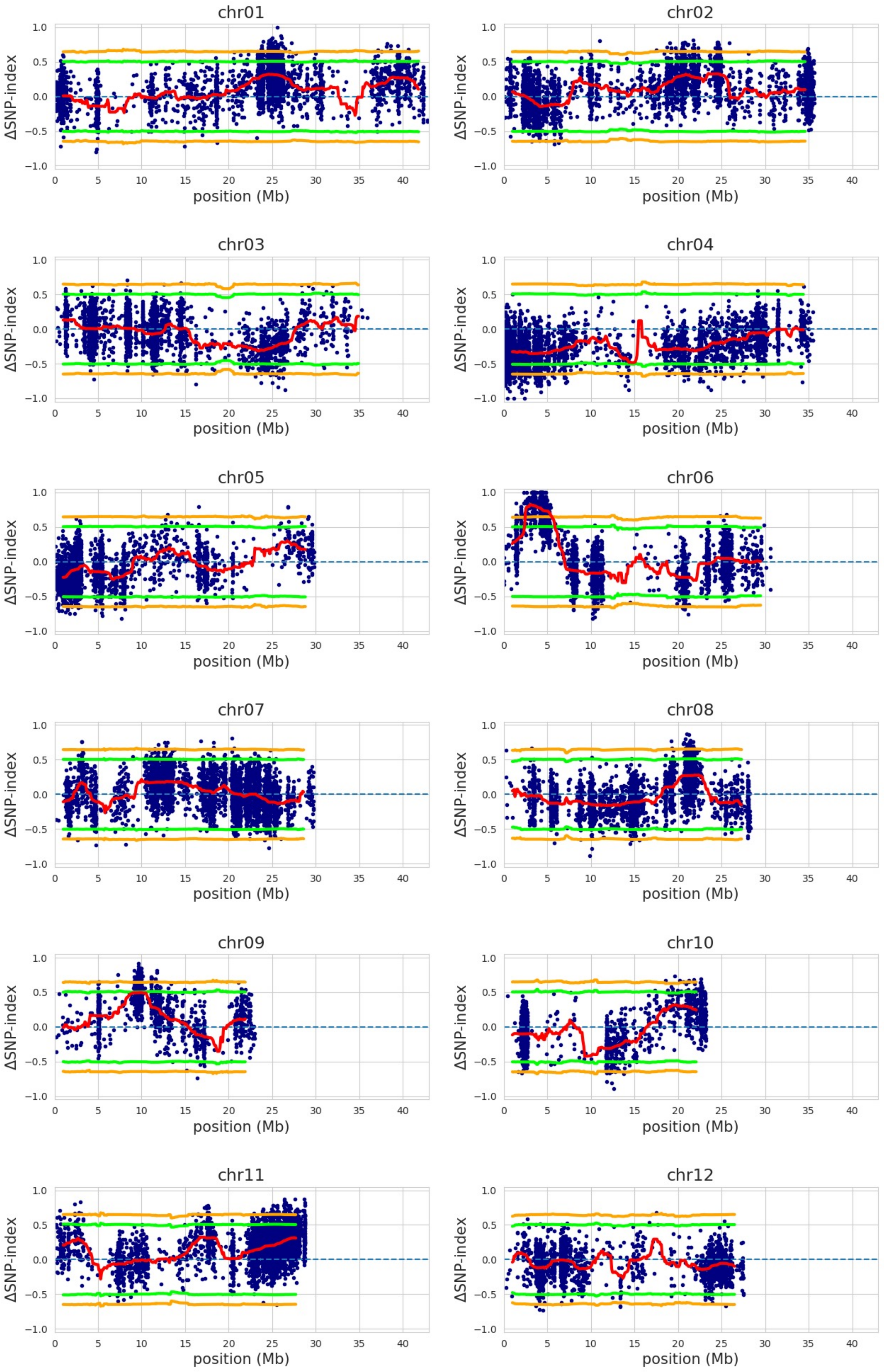

Supplemental Figure S1 (F) QTL-seq plot of RILs from QTL-seq v2.2.2.
The ΔSNP-index plot obtained by subtraction of the susceptible-bulk SNP-index from the resistance-bulk SNP-index for RILs obtained from a cross between Nortai and Hitomebore. Statistical confidence intervals under the null hypothesis of no QTL are shown (green: $P < 0.05$; yellow: $P < 0.01$).

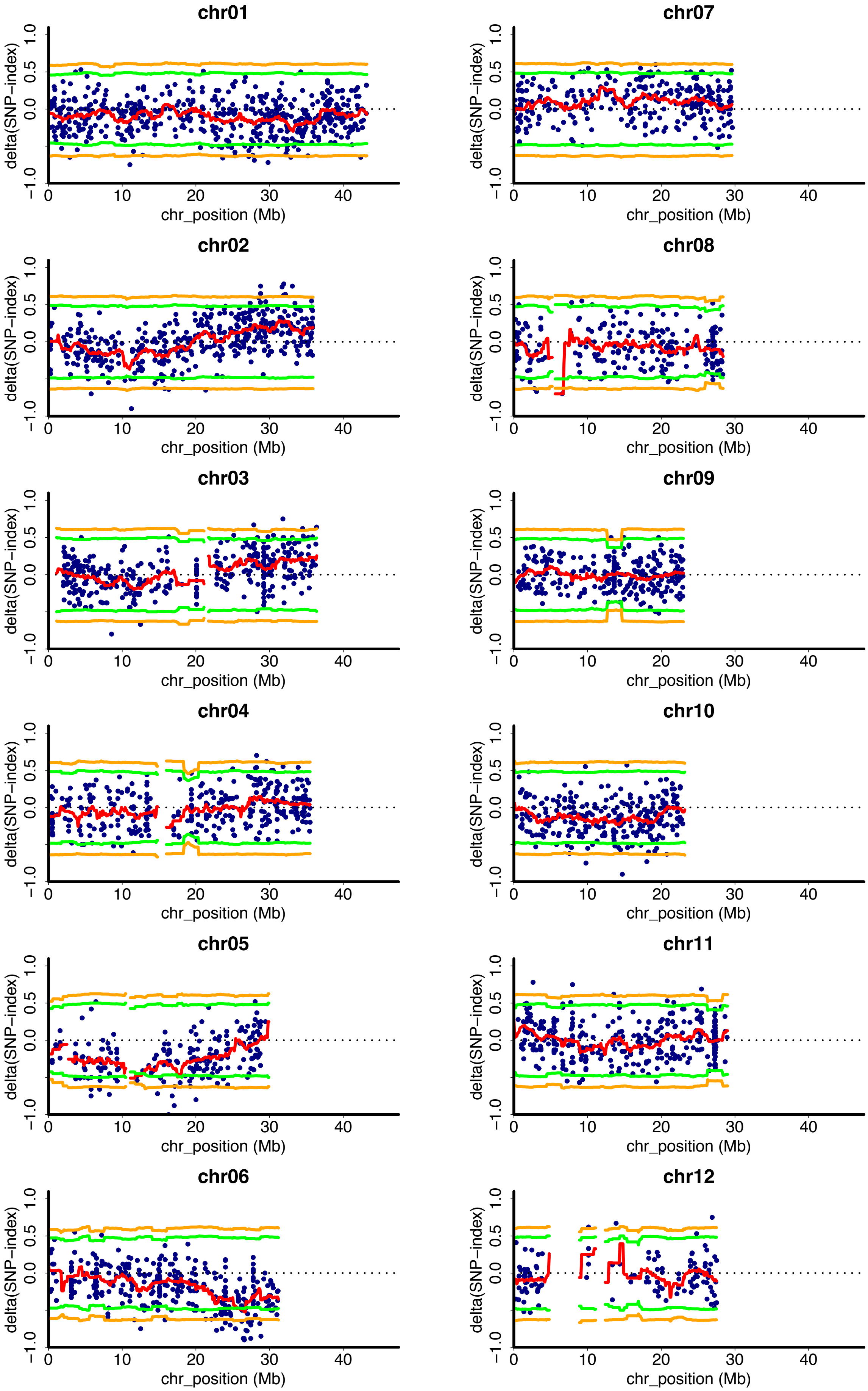

Supplemental Figure S1 (G) QTL-seq plot of F2 progeny from QTL-seq v1.4.5.
The ΔSNP-index plot obtained by subtraction of Highest-bulk SNP-index from Lowest-bulk SNP-index for F2 progeny obtained from a cross between Hitomebore and WRC57. Statistical confidence intervals under the null hypothesis of no QTL are shown (green: $P < 0.05$; yellow: $P < 0.01$).

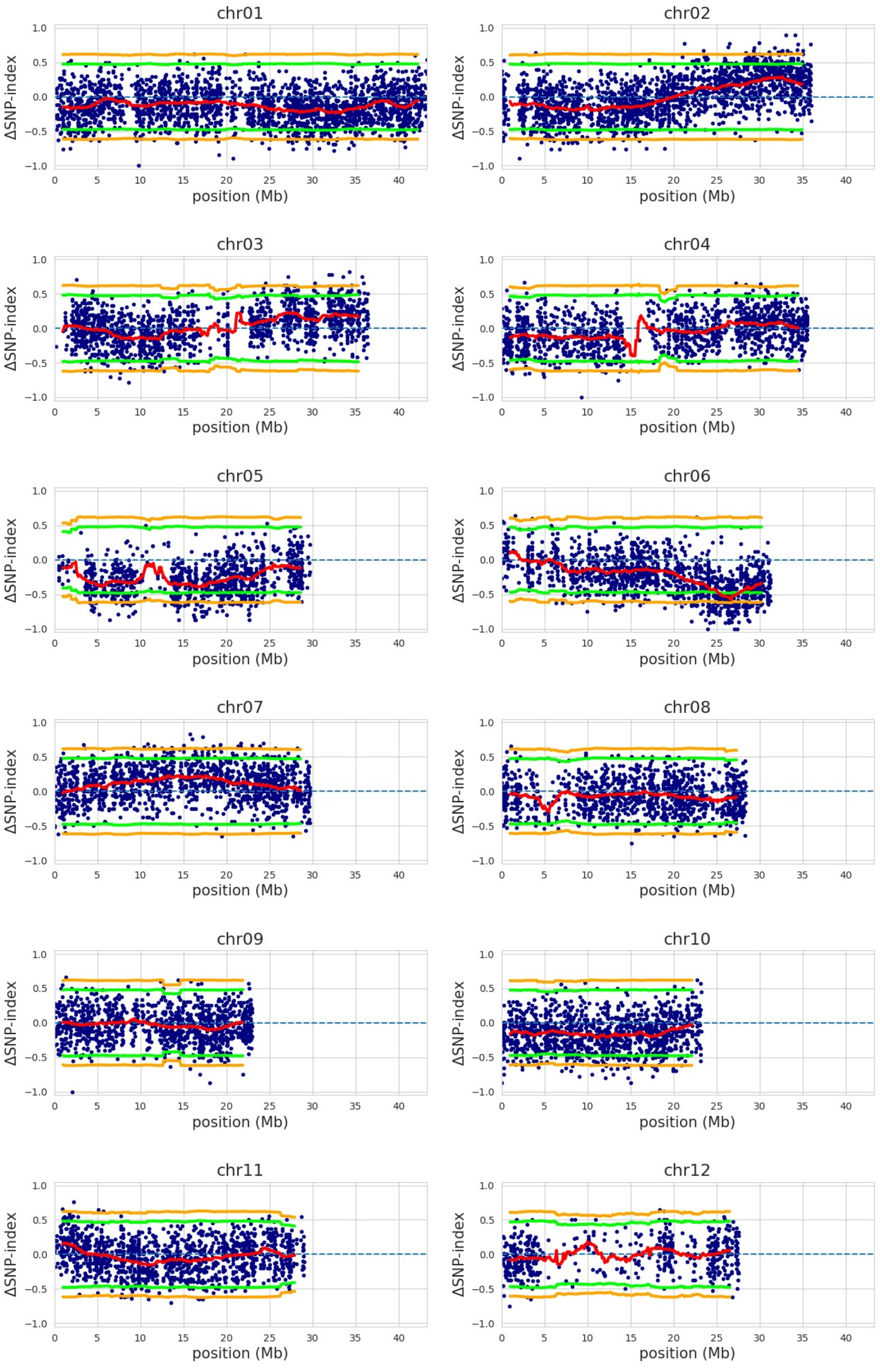

Supplemental Figure S1 (H) QTL-seq plot of F2 progeny from QTL-seq v2.2.2. The ΔSNP-index plot obtained by subtraction of Highest-bulk SNP-index from Lowest-bulk SNP-index for F2 progeny obtained from a cross between Hitomebore and WRC57. Statistical confidence intervals under the null hypothesis of no QTL are shown (green: $P < 0.05$; yellow: $P < 0.01$).
